# Structured-Light Sensor Using Two Laser Stripes for 3D Reconstruction without Vibrations

**DOI:** 10.3390/s141120041

**Published:** 2014-10-24

**Authors:** Rubén Usamentiaga, Julio Molleda, Daniel F. Garcia

**Affiliations:** Department of Computer Science and Engineering, University of Oviedo, Campus de Viesques, Gijón 33204, Asturias, Spain; E-Mails: jmolleda@uniovi.es (J.M.); dfgarcia@uniovi.es (D.F.G.)

**Keywords:** structured light, calibration, 3D reconstruction, vibrations

## Abstract

3D reconstruction based on laser light projection is a well-known method that generally provides accurate results. However, when this method is used for inspection in uncontrolled environments, it is greatly affected by vibrations. This paper presents a structured-light sensor based on two laser stripes that provides a 3D reconstruction without vibrations. Using more than one laser stripe provides redundant information than is used to compensate for the vibrations. This work also proposes an accurate calibration process for the sensor based on standard calibration plates. A series of experiments are performed to evaluate the proposed method using a mechanical device that simulates vibrations. Results show excellent performance, with very good accuracy.

## 1. Introduction

Surface reconstruction is one of the fundamental topics in computer vision. It has been applied in many different fields. Some recent examples include industrial inspection [1–3], cultural heritage [4], dental health care [5] or object recognition [6]. One of the most widely-used techniques is based on the projection of structured light [7]. The deformation of the projected pattern on the object is used to construct the 3D model of the object.

Laser light projection is considered one of the most reliable techniques for 3D reconstruction, with very good resolution, accuracy, and speed [8]. The most widely-used approach is based on the projection of a single laser plane over an object, forming a laser stripe. The projection is deformed according to the shape of the object. Thus, it is possible to extract a height profile from the projection. This technique requires a movement between the camera and laser projector, and the object. In this way, a set of height profiles can be used to reconstruct the whole surface of the object. For example, this technique is used for objects that move forward along a track while the camera and laser projector stay at a fixed position.

One problem with 3D reconstruction based on a single laser projector is vibrations. Up and down movements of the object while it moves forward along a track are interpreted as height variations, leading to an incorrect reconstruction of the surface of the object. Thus, the reconstruction of a flat object with vibrations results in a curved surface. Different filter strategies have been proposed to solve this problem. However, they can only be applied when assumptions can be made about the shape of the object or about the types of vibrations. For example, when vibrations are known to be at a specific frequency, a band-pass filter can be used to remove them [9]. When the shape of the object has some known features, such as straight lines connected by a curved corner [10], these can be detected and used to estimate and remove vibrations. However, when these invariants do not exist, these methods cannot be used.

In [11] a theoretical method to remove or reduce the effects of vibrations in 3D reconstruction using multiple laser stripes was proposed. Using more than one laser stripe provides additional information that can be used to estimate and remove vibrations. Height profiles are acquired multiple times for the same section of a moving object. Thus, variations between them can be detected. These variations do not depend on the shape of the object, but on vibrations. More than two laser stripes are required to estimate complex vibrations that contain a combination of vertical translations and rotations. However, tests indicate that a laser-based 3D reconstruction method using two laser stripes presents a far more cost efficient solution than other approaches, as well as having similar performance. The proposed procedure has not been tested in real working conditions, thus the conclusions are drawn based on simulated data.

A structured-light sensor using two laser stripes can provide an accurate 3D reconstruction without the effects of vibrations. However, this type of sensor requires a more complex calibration. In this case, in addition to calibrating the mapping from the 3D scene to the image, each laser plane must also be calibrated. Moreover, vibrations using multiple laser stripes are removed by detecting height variations between planes. Thus, a transformation between laser planes is also required. This work presents a structured light sensor using two laser stripes for 3D reconstruction without vibrations. The sensor is applied to real working conditions, where an accurate calibration process is proposed.

In 3D reconstruction using laser stripe projection, the measurement plane is the plane built by the projection of the laser light onto the object, which is called the laser plane. Thus, in order to measure objects that lie on the laser plane it is necessary to calibrate its position, that is, calibrate the extrinsic parameters (rotation and translation) from the laser plane to the camera [12]. The calibration procedure can be carried out using standard calibration plates or specific 3D objects of known dimensions. For example, a standard calibration plate can be placed exactly parallel to the laser plane [13,14]. However, it is very difficult to manually place the calibration pattern perfectly at this position. Moreover, small alignment deviations would lead to inaccuracies in the resulting calibration. Another approach is based on 3D objects of known dimensions [15–17]. The problem with this approach is that it generally requires a difficult-to-build calibration target, only valid for particular applications. The proposed procedure for the calibration of the laser plane used in this work is based on a standard calibration pattern. Thus, the calibration procedure is easier and does not require expensive and complex calibration objects or elaborate setups, while producing highly accurate results with no previous assumptions.

The calibration procedure is applied in three steps. First, the calibration pattern is observed in different positions. The acquired images are used to calibrate the intrinsic parameters of the camera using an accurate model that includes distortions. Then, the same calibration plate is used to calibrate the laser planes by projecting the laser in two different positions in the field of view of the camera. The final step is the transformation between laser planes, which is calculated using the same geometrical information obtained in the previous step. The proposed procedure is easy to apply, does not require special equipment, and provides very accurate results. Moreover, although it is applied to 3D reconstruction using two laser stripes, the procedure can be generalized for multiple laser stripes.

In order to test the proposed 3D reconstruction sensor using two laser stripes, different experiments have been carried out. The objective is to reconstruct the surface of an object that is affected by vibrations. These vibrations are simulated by placing the object on a mechanical device that moves vertically. The results show very good performance.

### 2. 3D Reconstruction Using Two Laser Stripes

3D reconstruction using one laser projector is based on the acquisition of height profiles as the object moves. In a different scenario, it is the laser projector that moves, for example on a robotic arm. In both cases, a relative movement between the projector and the object is required. Under optimal conditions, using a second laser projector redundantly provides the same height profiles as the first projector, but with a time offset depending on the distance between lasers and the speed of the movement. When vibrations affect the movement, height profiles vary and the redundant information provided by the two lasers can be used to estimate vibrations, and thus, to remove them.

**Figure 1.**
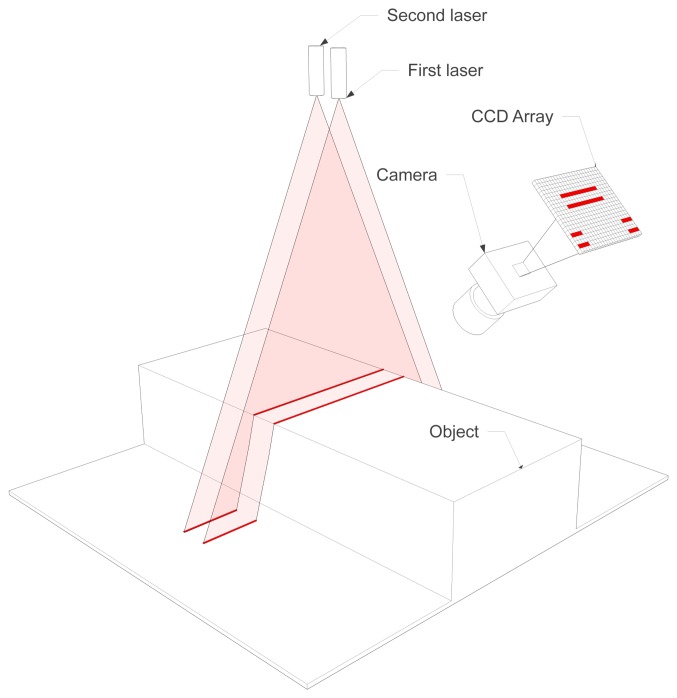
3D reconstruction architecture using two laser stripes.

The architecture can be seen in Figure 1. The first and second lasers project a parallel laser stripe onto the object. The projections of the laser stripes onto the object are very close. Thus, the movement of the object caused by vibrations affects the deformation of the laser stripes equally. When the object moves forward, the first laser stripe is projected on the same section of the object as previously the second laser stripe (depending on the direction of the movement of the object it could be the opposite). As both laser stripes are projected on the same section of the object, the resulting height profiles must be identical. Any possible difference is caused solely by vibrations. Therefore, this redundant information can be used to estimate and remove vibrations.

Vibrations modify the position of points in space, based on different movements. Therefore, vibrations can be modeled based on geometrical transformations. Complex vibrations include translations and rotations. Thus, they can be modeled using Equation (1), which is a transformation obtained by composing a translation and a rotation [18]. The parameters of this transformation are the vertical translation (*t_y_*), the rotation angle (*θ*), and the pivot point of the rotation (*x_p_*,*y_p_*).

(1) $$\text{V}=\text{R}\cdot \text{T}=\left(\begin{array}{ccc} \cos(\theta ) & -\sin(\theta ) & x_{p}\cos(\theta )-(t_{y}-y_{p})\sin(\theta )+x_{p}\\ \sin(\theta ) & \cos(\theta ) & -x_{p}\sin(\theta )+(t_{y}-y_{p})\cos(\theta )+y_{p}\\ 0 & 0 & 1 \end{array}\right)$$

The model is parametrized by four values: *θ*, *x_p_*, *y_p_*, and *t_y_*. In order to calculate these four values, four equations are needed. These four equations can be created from two points acquired twice, that is, from three laser stripes.

The most common type of vibrations consists of vertical translations only. This type of vibration can be modeled using Equation (2).

(2) $$\text{V}=\text{T}=\left(\begin{array}{ccc} 1 & 0 & 0\\ 0 & 1 & t_{y}\\ 0 & 0 & 1 \end{array}\right)$$

Using two laser stripes, the height of a point on the object is calculated twice, once on the second laser stripe (*S*) and once on the first laser stripe (*F*) as the object moves forward. Thus, the vertical translation between these two points can be calculated using Equation (3).

(3) $$t_{y}=S_{y}-F_{y}$$

Using two laser stripes, only translations can be estimated. Using three laser stripes, both translations and rotations can be estimated. An overdetermined system is produced with more than three laser stripes, which delivers a more robust solution. However, a system in which only two laser stripes are used produces very low error even under complex vibrations with rotations. Moreover, a system with three laser stripes is more sensitive to noise, since adding noise can provoke spurious estimations of the vibrations [11], resulting in outliers in the reconstruction of the shape. A third laser projector also increases the cost and the maintenance of a 3D reconstruction system.

## 3. Calibration

### 3.1. Camera Calibration

Camera calibration is a required step in 3D reconstruction to extract metric information from 2D images [18]. The objective is to determine a set of parameters that describe the mapping between 3D points in the world coordinate system and the 2D image coordinates. The overall performance of 3D reconstruction strongly depends on the accuracy of the camera calibration [19].

**Figure 2.**
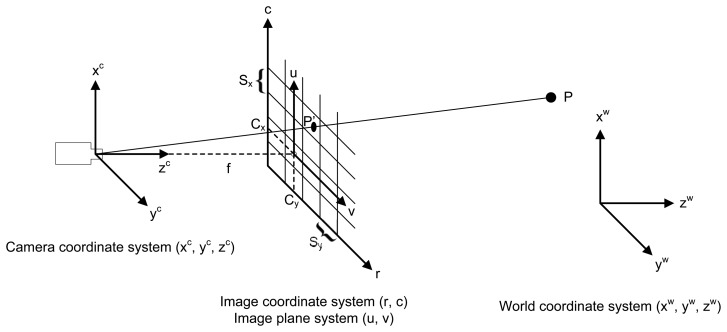
Camera model.

The perspective projection of the world coordinates onto the image is generally modeled using the pinhole camera model. Figure 2 shows a graphical representation of this model. Using this model, the image of a 3D point, *P*, is formed by an optical ray passing through the optical center and intersecting the image plane. The result is the point *P′* in the image plane, which is located at a distance *f* (focal length) behind the optical center. In Figure 2 the image plane is positioned between the scene point and the optical center, which is mathematically equivalent to considering the image plane behind the optical center. This approach is generally used because in this way the image coordinate system is aligned with the pixel coordinate system.

The first step to mathematically describe the projection of 3D points on the 2D image plane is the transformation from the world coordinate system (WCS) to the camera coordinate system (CCS), i.e., *w* → *c*. The transformation from WCS to CCS is given by Equation (4). Using this equation, the camera coordinates of a point *P^c^* = (*x^c^*, *y^c^*, *z^c^*)*^T^* are calculated from its world coordinate *P^w^* = (*x^w^*, *y^w^*, *z^w^*)*^T^*using the rigid transformation *H_w_*_→_*_c_*.

(4) $$\left(\begin{array}{c} P^{c}\\ 1 \end{array}\right)={\text{H}}_{\text{w}\to \text{c}}\left(\begin{array}{c} P^{w}\\ 1 \end{array}\right)$$

The transformation from the WCS to the CCS is performed using the homogeneous transformation matrix *H_w_*_→_*_c_*, which relates the WCS to the CCS. *H_w_*_→_*_c_* includes three translations (*t_x_*, *t_y_*, *t_z_*) and three rotations (*α*, *β*, *γ*). These six parameters are called the extrinsic camera parameters, and describe the rotation (*R_w_*_→_*_c_*) and translation (*t_w_*_→_*_c_*) from the WCS to the CCS. Thus, Equation (4) can also be expressed as Equation (5).

(5) $$\left(\begin{array}{c} x^{c}\\ y^{c}\\ z^{c}\\ 1 \end{array}\right)=\left(\begin{array}{cc} {\text{R}}_{\text{w}\to \text{c}} & {\text{t}}_{\text{w}\to \text{c}}\\ 0 0 0 & 1 \end{array}\right)\left(\begin{array}{c} x^{w}\\ y^{w}\\ z^{w}\\ 1 \end{array}\right)$$

Based on the pinhole model, the projection of the point in the CCS onto the image coordinate system is calculated using Equation (6).

(6) $$\left(\begin{array}{c} \text{u}\\ \text{v} \end{array}\right)=\frac{\text{f}}{z^{c}}\left(\begin{array}{c} x^{c}\\ y^{c} \end{array}\right)$$

The pinhole model is only an ideal approximation of the real camera projection. Imaging devices introduce a certain amount of nonlinear distortion [20]. Thus, when high accuracy is required, lens distortion must be taken into account [21,22]. One of the most accurate methods to model lens distortion is the polynomial model [23]. Using this model, three parameters are used to model radial distortion (*k*_1_, *k*_2_, *k*_3_), and two to model decentering distortion (*p*_1_, *p*_2_). The distorted image plane coordinates are transformed into undistorted image plane coordinates using Equations (7) and (8), where $\text{r}=\sqrt{{\tilde{\text{u}}}^{2}+{\tilde{\text{v}}}^{2}}$.

(7) $$\text{u}=\tilde{\text{u}}+\tilde{\text{u}}({\text{k}}_{1}{\text{r}}^{2}+{\text{k}}_{2}{\text{r}}^{4}+{\text{k}}_{3}{\text{r}}^{6})+2{\text{p}}_{1}\tilde{\text{u}}\tilde{\text{v}}+{\text{p}}_{2}({\text{r}}^{2}+2{\tilde{\text{u}}}^{2})$$

(8) $$\text{u}=\tilde{\text{u}}+\tilde{\text{v}}({\text{k}}_{1}{\text{r}}^{2}+{\text{k}}_{2}{\text{r}}^{4}+{\text{k}}_{3}{\text{r}}^{6})+2{\text{p}}_{2}\tilde{\text{u}}\tilde{\text{v}}+{\text{p}}_{1}({\text{r}}^{2}+2{\tilde{\text{v}}}^{2})$$

The final step is the transformation from the image plane coordinate system to the image coordinate system, that is, the pixel coordinate system. This transformation is achieved using Equation (9), where *S_x_* and *S_y_* are scaling factors that represent the horizontal and vertical distances between the sensor elements on the CCD chip of the camera and the point (*Cx*, *Cy*)*^T^*, which is the perpendicular projection of the optical center onto the image plane.

(9) $$\left(\begin{array}{c} \text{r}\\ \text{c} \end{array}\right)=\left(\begin{array}{c} \frac{\tilde{\text{v}}}{S_{y}}+C_{y}\\ \frac{\tilde{\text{u}}}{S_{x}}+C_{x} \end{array}\right)$$

The ten parameters *I_c_* = (*f*,*k*_1_,*k*_2_,*k*_3_,*p*_1_,*p*_1_,*S_x_*,*S_y_*,*C_x_*,*C_y_*) are called the intrinsic camera parameters, as they describe how the camera projects 3D points onto 2D image coordinates. The number of intrinsic camera parameters depends on the lens distortion model used; other models require a different number of parameters.

In order to determine the optimal values for both the extrinsic and intrinsic camera parameters, image observations of a known target are required. There are various possible alternatives. Early procedures were based on a calibration object whose geometry in 3D space is known with high precision [24]. The problem with this approach is that it requires an expensive calibration object and a complicated setup. An alternative approach is based on a planar pattern observed at different orientations without previous knowledge of the motion of the camera or the calibration object [25]. The calibration pattern is easier to build and the calibration setup is much simpler.

**Figure 3.**
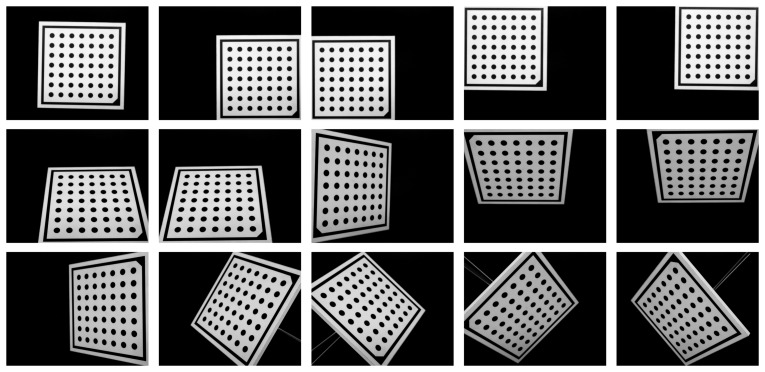
Images of the planar pattern at different orientations.

Figure 3 shows images of the planar pattern at different orientations. The polynomial model requires a complete coverage of the image in order to produce accurate results. Otherwise, distortions may be modeled inaccurately.

**Figure 4.**
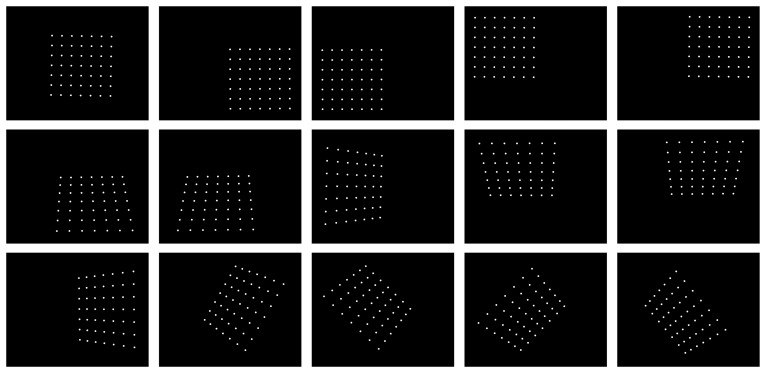
Centers of the circles in the calibration pattern.

Feature extraction from the images provides the position of known points in the calibration pattern. Using this particular pattern, the centers of the circles are calculated, as can be seen in Figure 4. These points are calculated using a combination of Gaussian filtering, thresholding, contour processing, and edge detection with sub-pixel accuracy. Based on these observations and the previous knowledge of the positions of the circles in the pattern, the camera parameters are calculated. In order to achieve accurate results, a homogeneous distribution of calibration pattern within the field of view of the camera is required.

The mathematical procedure to accurately determine the values of the extrinsic and intrinsic camera calibration is complex and requires a combination of closed-form solution and linear least-squares [26]. Finally, all the parameters are refined by minimizing Equation (10), where *M* is the mapping of point ${\text{P}}_{\text{j}}^{\text{w}}$ in image *i* according to the previous equations, and ${\text{P}}_{\text{j}}^{\text{I}}$ is the point in image coordinates. This final step is a nonlinear minimization problem, which is solved with the Levenberg-Marquardt algorithm [27]. The result of this process is a very accurate estimation of the intrinsic and extrinsic parameters of the camera. The intrinsic parameters describe the internal projection of the points in the camera. Thus, the obtained values remain valid only as long as the camera and lens maintain their configuration. The extrinsic parameters are specific for each orientation of the planar calibration pattern, as they describe the pose from the world (the plane where the calibration pattern is placed) to the camera.

(10) $$\sum_{\text{i}=1}^{\text{n}}\sum_{\text{j}=1}^{\text{m}}\left\|{\text{p}}_{\text{ij}}^{\text{I}}-\text{M}({\text{H}}_{\text{w}\to \text{c}},I_{c},{\text{P}}_{\text{j}}^{\text{w}})\right\|$$

After the calibration, the mapping from any point in the world *P^w^* to the image coordinate *p^I^* is carried out using Equation (11). The inverse mapping is obtained with Equation (12).

(11) $$p^{I}=\text{M}({\text{H}}_{\text{w}\to \text{c}},I_{c},P^{w})$$

(12) $$P^{w}={\text{M}}^{-1}({\text{H}}_{\text{w}\to \text{c}},I_{c},P^{I})$$

### 3.2. Laser Plane Calibration

The camera model provides the mapping of 3D points in world coordinates to 2D image coordinates. However, the inverse transformation requires additional knowledge about the scene, in particular, the position of the measurement plane. With this information, the inverse transformation can be solved by intersecting an optical ray with this plane. The measurement plane is calibrated with the extrinsic camera parameters that indicate the transformation from the WCS (measurement plane) to the CCS. In 3D reconstruction using laser stripe projection, the measurement plane is the plane built by the projection of the laser light onto the object, which is called the laser plane.

The laser plane calibration requires a fixed position for the camera and for the laser projector. Then, the calibration pattern is placed in two different positions in the field of view of the camera where the laser stripe is projected: Position 1 and position 2. For each of these positions two images are acquired: One where the calibration pattern is observed and one where the laser stripe projected onto the calibration pattern is observed. When the first image is acquired, the laser projector is turned off and the exposure time of the camera is adjusted in order to observe the calibration circles correctly. For the second image, the laser projector is turned on and the exposure time of the camera is decreased. This way, only the projection of the laser stripe onto the calibration pattern is observed by the camera. The calibration pattern is then translated or tilted and the procedure is repeated.

**Figure 5.**
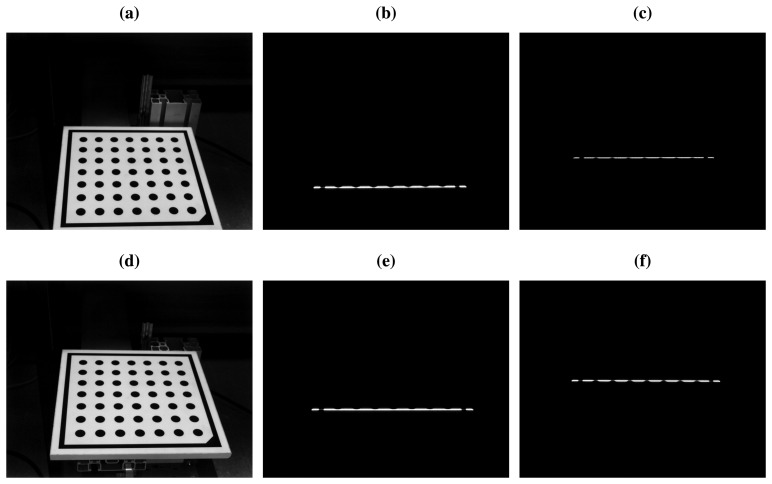
Images used for the calibration of the laser planes. (**a**) Calibration pattern at position 1; (**b**) Projection of the first laser on the calibration pattern at position 1; (**c**) Projection of the second laser on the calibration pattern at position 1; (**d**) Calibration pattern at position 2; (**e**) Projection of the first laser on the calibration pattern at position 2; (**f**) Projection of the second laser on the calibration pattern at position 2.

The calibration of the laser plane is the same for the first and for the second laser. Thus, in order to simplify the procedure, the second laser plane is calibrated at the same time as the first. The only difference is that a third image needs to be acquired for each of the two positions of the calibration pattern. This third image is acquired with the second laser projector turned on and the first turned off. This way, only the projection of the second laser stripe onto the calibration pattern is observed by the camera. Finally a set of six images are acquired, as can be seen in Figure 5. In this case, the calibration pattern is translated upwards from position 1 to position 2.

A possible simplification for the calibration of the two laser planes would be to obtain a single image with the projection of the two laser stripes at the same time. They could be distinguished based on their position in the image: The one below is the first and the one above is the second. In a real scenario during measurement, with concave or convex changes parallel to the laser stripe, the projected lines acquired by the camera may be disconnected leading to an incorrect order of the stripes. This would require a robust laser stripe extraction algorithm to decide which section belongs to which laser stripe. This algorithm could be based on previous information about where each laser stripe was, and stripe continuity.

Images of the calibration pattern at positions 1 and 2 (Figure 5a,d) can be used for camera calibration. They can be used to increase the accuracy of the estimation of the intrinsic parameters, but more importantly, the extrinsic parameters that describe the orientation of the planar calibration pattern are obtained. Therefore, for each of these two images the transformation matrix that relates the WCS to the CCS is obtained. For the image of the calibration pattern at position 1 (world 1), the transformation *H_w_*__1_→_*_c_* is obtained. Similarly, *H_w_*__2_→_*_c_* is obtained from the image of the calibration pattern at position 2 (world 2).

**Figure 6.**
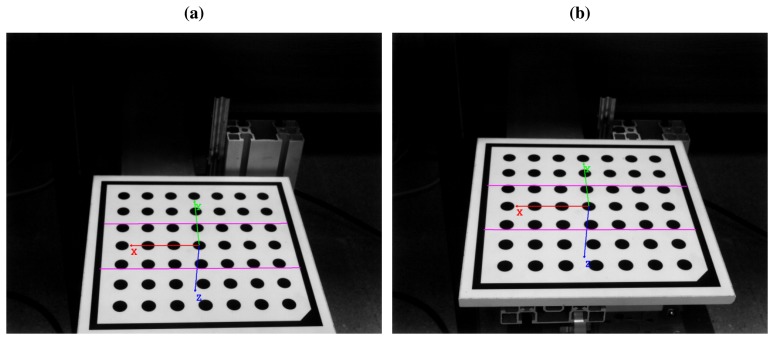
World coordinates and extracted laser stripes. (**a**) World coordinate when calibration pattern is at position 1 (*w*_1_) and first and second laser stripes (*f*_1_ and *s*_1_); (**b**) World coordinate when calibration pattern is at position 2 (*w*_2_) and first and second laser stripes (*f*_2_ and *s*_2_).

The images where the laser stripes are projected onto the calibration pattern are now processed (Figure 5b-f). The goal is to extract the laser stripe coordinates from the images. Depending on the application and the environment, different methods can be used [28]. In general applications, a method based on Gaussian filtering and edge detection provides good results [29]. Regardless of the laser stripe extraction method, for each image a set of points with the image coordinates of the laser projection onto the calibration pattern is obtained. The first (*f*) laser stripe extracted from the calibration pattern at position 1 (Figure 5b) will be called *f*_1_, and *f*_2_ when it is at position 2 (Figure 5e). A similar process is applied to the second laser (*s*), resulting in *s*_1_ (Figure 5c) and *s*_2_ (Figure 5f). The extracted laser stripes are in image coordinates. Figure 6 shows the axis of the reference system for world coordinates at positions 1 and 2, and the positions of the first and second laser stripes.

The corresponding world coordinates can be obtained using Equations (13)—(16). Laser stripes *f*_1_ and *s*_1_ are extracted from the calibration pattern at position 1, thus, they are translated to the coordinates of this world reference (*w*_1_). Laser stripes *f*_2_ and *s*_2_ are similarly translated to *w*_2_. The result is $F_{i}^{wj}$, the coordinates of the first laser in the coordinates of world *j* when it is projected onto the laser stripe at position *i*, and the equivalent for $S_{i}^{wj}$. In all cases, the coordinate *z* is zero, as all points lie on the measurement plane.

(13) $${\text{F}}_{1}^{\text{w}1}={\text{M}}^{-1}({\text{H}}_{\text{w}1\to \text{c}},I_{c},{\text{f}}_{1})$$

(14) $${\text{F}}_{2}^{\text{w}2}={\text{M}}^{-1}({\text{H}}_{\text{w}2\to \text{c}},I_{c},{\text{f}}_{2})$$

(15) $${\text{S}}_{1}^{\text{w}1}={\text{M}}^{-1}({\text{H}}_{\text{w}1\to \text{c}},I_{c},{\text{s}}_{1})$$

(16) $${\text{S}}_{2}^{\text{w}2}={\text{M}}^{-1}({\text{H}}_{\text{w}2\to \text{c}},I_{c},{\text{s}}_{2})$$

The next step is to transform world coordinates so that they all share the same reference system. The rigid transformation *H_w_*__1_→_*_c_* can be inverted using Equation (17). Then, a composition of transformations can be applied to obtain the transformation from *w*_2_ to *w*_1_, as seen in Equation (18).

(17) $${\text{H}}_{\text{c}\to \text{w}1}={\text{H}}_{\text{w}1\to \text{c}}^{-1}$$

(18) $${\text{H}}_{\text{w}2\to \text{w}1}={\text{H}}_{\text{c}\to \text{w}1}{\text{H}}_{\text{w}2\to \text{c}}$$

The coordinates of the laser stripes can be transformed to the same reference system (*w*_1_) using Equations (19) and (20).

(19) $${\text{F}}_{2}^{\text{w}1}={\text{H}}_{\text{w}2\to \text{w}1}{\text{F}}_{2}^{\text{w}2}$$

(20) $${\text{S}}_{2}^{\text{w}1}={\text{H}}_{\text{w}2\to \text{w}1}{\text{S}}_{2}^{\text{w}2}$$

**Figure 7.**
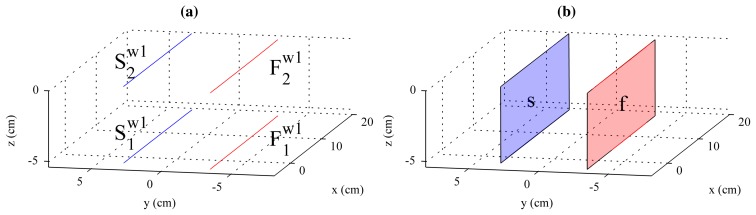
Laser planes fitting. (**a**) World coordinate of the laser stripes (*w*_1_); (**b**) Plane of the first (*f*) and second lasers (*s*).

Figure 7a shows all the laser stripes in the same world coordinates. As can be seen, ${\text{F}}_{1}^{\text{w}1}$ and ${\text{F}}_{2}^{\text{w}1}$ lie on the plane of the first laser, and ${\text{S}}_{1}^{\text{w}1}$ and ${\text{S}}_{2}^{\text{w}1}$ lie on the plane of the second laser. Thus, they can be used to fit a plane that mathematically describes the laser plane. All the points in ${\text{F}}_{1}^{\text{w}1}$ and ${\text{F}}_{2}^{\text{w}1}$ are used to fit plane *f*, and all the points in ${\text{S}}_{1}^{\text{w}1}$ and ${\text{S}}_{2}^{\text{w}1}$ are used to fit plane *s*. The result can be seen in Figure 7b. Plane fitting is a least squares problem that can be solved very accurately using singular value decomposition (SVD) [30].

Plane fitting provides the coordinates of laser plane *f* and laser plane *s* with respect to *w*_1_. Each plane is described with the normal vector to the plane. The transformation from the laser planes to *w*_1_ can be calculated by aligning the normal vectors of the planes to the reference system using rotations. The results are *H_f_*_→_*_w_*__1__ and *H_s_*_→_*_w_*__1__, which describe the transformation from the first and second laser to *w*_1_, respectively.

The final extrinsic camera parameters for the first plane, *H_f_*_→_*_c_*, can be calculated using Equation (21). Similarly, Equation (22) is used for the second laser. *H_f_*_→_*_c_* and *H_s_*_→_*_c_* relate the WCS for each laser to the CCS.

(21) $${\text{H}}_{\text{f}\to \text{c}}={\text{H}}_{\text{w}1\to \text{c}}{\text{H}}_{\text{f}\to \text{w}1}$$

(22) $${\text{H}}_{\text{s}\to \text{c}}={\text{H}}_{\text{w}1\to \text{c}}{\text{H}}_{\text{s}\to \text{w}1}$$

**Figure 8.**
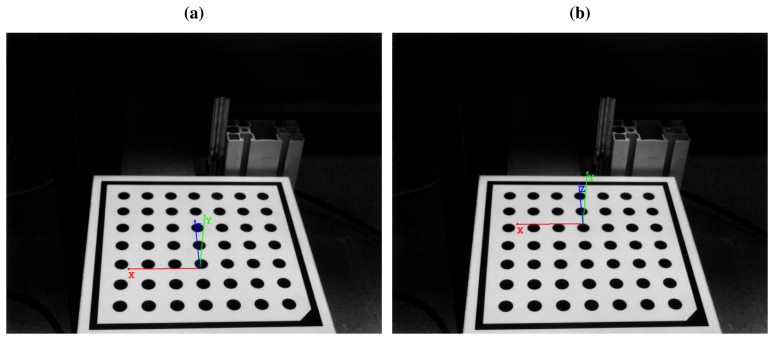
World reference systems for the two lasers. (**a**) World reference system for the first laser; (**b**) World reference system for the second laser.

*H_f_*_→_*_c_* and *H_s_*_→_*_c_* are the extrinsic camera parameters required to extract metric information from 2D images on the laser planes. Figure 8 shows the world reference systems for the two lasers.

### 3.3. Transformation Between Planes

3D reconstruction using two laser planes uses the redundant information on the second plane to compensate for vibrations in the first plane. Therefore, it is necessary to transform the coordinates from the second plane to the first plane.

A laser stripe *s*, extracted from the second laser, is translated to world coordinates *S^s^* using Equation (23). The coordinates are with respect to *s*, the second laser plane.

(23) $$S^{s}={\text{M}}^{-1}({\text{H}}_{\text{s}\to \text{c}},I_{c},\text{s})$$

The transformation from *s* to *f* can be obtained using a composition of transformations, as seen in Equation (24), where *H_c_*_→_*_f_* is equal to ${\text{H}}_{\text{f}\to \text{c}}^{-1}$.

(24) $${\text{H}}_{\text{s}\to \text{f}}={\text{H}}_{\text{c}\to \text{f}}{\text{H}}_{\text{s}\to \text{c}}$$

The laser stripe extracted from the second laser is transformed to the world coordinates of the first laser using Equation (25).

(25) $$S^{f}={\text{H}}_{\text{s}\to \text{f}}S^{s}$$

### 3.4. Summary and Generalization for Multiple Laser Stripes

**Figure 9.**
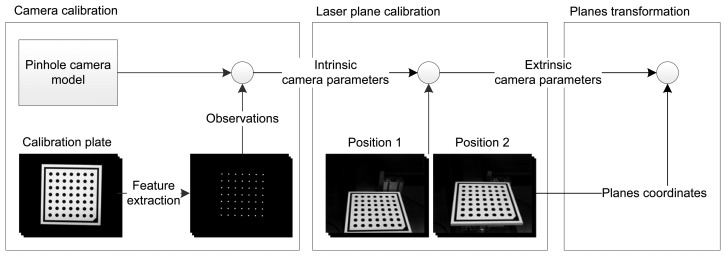
Summary of the calibration procedure.

Figure 9 shows a summary of the steps of the calibration process. The proposed calibration procedure can be easily extended for multiple laser stripes. The summary of the general procedure is as follows:

1. The camera is adjusted for the measurement, including focus, zoom, diaphragm, etc. Then, a planar calibration pattern is placed in different poses in the field of view of the camera. For each pose, an image is acquired. The coordinates of the markers in the calibration pattern are used to calibrate the intrinsic parameters of the camera using the procedure described in Section 3.1.
2. The calibration pattern is placed in two different positions in the field of view of the camera where the laser stripes are projected. For each of these positions two images are acquired: One where the calibration pattern is observed, and one where all the laser stripes projected onto the calibration pattern are observed. These four images are used to calibrate the extrinsic parameters of the camera for each laser using the procedure described in Section 3.2. The laser stripe extraction must identify and tag the extracted laser stripes to carry out the calibration.
3. Finally, the transformation from each laser plane to one common plane is calculated to obtain all metric information in the same reference system using the procedure described in Section 3.3.

## 4. Results and Discussion

In order to test the 3D reconstruction procedure using two laser stripes, the proposed procedure has been applied to a real test case. The objective is the reconstruction of two different surfaces placed on a mechanical device that moves up and down. This movement simulates vertical translation, the most common type of vibration.

**Figure 10.**
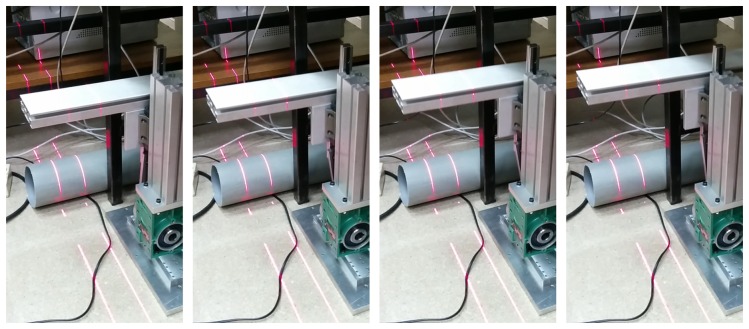
Device used to simulated vibrations while it moves the flat platform upwards.

Figure 10 shows the device that simulates vibrations. The device moves a flat surface up and down, parallel to the laser planes. Two laser stripes are projected onto the moving surface and a camera acquires images from the projection. From one image to the next, the surface onto which the laser stripes are projected changes its vertical position. In the prototype, this vertical translation is considered a vibration. No other movements affect the surface onto which the laser stripes are projected. Thus, the movement on the *Z* axis (according to Figure 8) is simulated by considering that the strip is moving forward at a constant speed, that is, from one image to the next there has been a constant movement perpendicular to the laser plane. In real cases, speed can be variable. This requires accurate measurements of the speed so that height profiles can be aligned from different images. The shape of the surface onto which the laser stripes are projected is constant. Thus, from one image to the next, vibrations are the sole cause of changes in the position of the laser stripe in the images.

Calibration was carried out using the proposed procedure. The internal camera calibration was performed using the images shown in Figure 3. The camera used in the experiments is the Mikrotron MC1364, which uses a CMOS sensor with a resolution of 1024 × 1280. The values obtained for the intrinsic parameters are shown in Table 1. The resulting calibration error was 0.140 pixels.

The external calibration was performed placing the calibration pattern on the device used to simulate vibrations. Position 1 was at the lowest vertical position of the device, and position 2 was at the highest vertical position of the device. Images of these two positions and the laser projections are seen in Figure 5, and the calculated planes are shown in Figure 7. The results of the laser planes calibration are the transformation matrices Equations (26) and (27), which describe the rigid transformations from the laser planes to the camera. The resulting transformation between planes is Equation (28).

(26) $${\text{H}}_{\text{f}\to \text{c}}=\left(\begin{array}{cccc} -0.9961 & 0.0604 & -0.0637 & 0.0083\\ -0.0074 & -0.7807 & -0.6248 & 0.0709\\ -0.0875 & -0.6220 & 0.7781 & 0.5740\\ 0 & 0 & 0 & 1 \end{array}\right)$$

(27) $${\text{H}}_{\text{s}\to \text{c}}=\left(\begin{array}{cccc} -0.9959 & 0.0607 & -0.0668 & 0.0052\\ -0.0053 & -0.7786 & -0.6275 & 0.0313\\ -0.0901 & -0.6246 & 0.7758 & 0.6249\\ 0 & 0 & 0 & 1 \end{array}\right)$$

(28) $${\text{H}}_{\text{s}\to \text{f}}=\left(\begin{array}{cccc} 1.0000 & -0.0000 & 0.0034 & -0.0011\\ -0.0000 & 1.0000 & 0.0033 & -0.0009\\ -0.0034 & -0.0033 & 1.0000 & 0.0645\\ 0 & 0 & 0 & 1 \end{array}\right)$$

**Figure 11.**
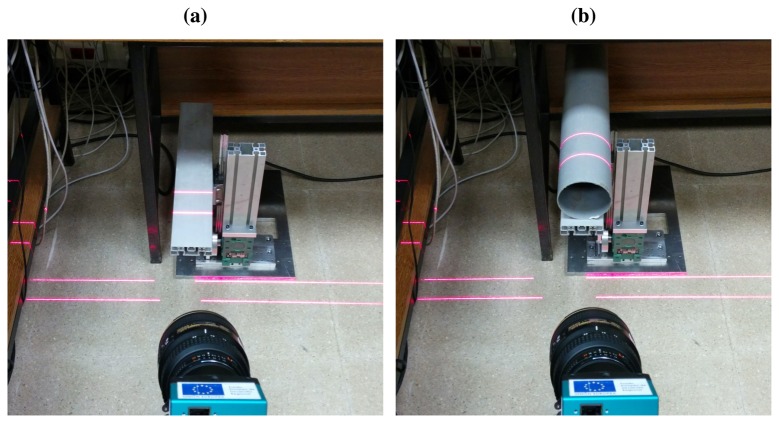
Surfaces used in the experiments. (**a**) Flat surface; (**b**) Curved surface.

Two different surfaces are used, one flat and one curved. The flat surface is part of the device used to simulate vibrations itself. The curved surface is obtained from a PVC pipeline of 110 mm diameter placed on the device. Images of the projection of the laser onto these two surfaces are shown in Figure 11.

**Figure 12.**
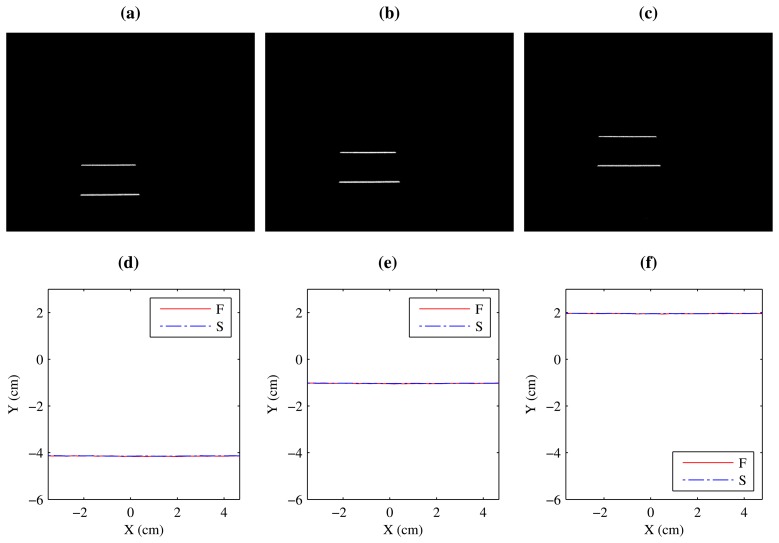
Projection of the laser stripes onto the flat surface. (**a–c**) Image coordinates; (**d–f**) World coordinates.

Figure 12a–c show images of the projection of the two laser stripes onto the flat surface while it is moving upwards. The two stripes seem to have different lengths due to the perspective projection of the camera. However, when the laser stripes are converted to world coordinates, they are identical, as can be seen in Figures 12d–f. In this case, the two laser stripes have been converted to the same reference system.

Accurate laser alignment is critical in order to estimate vibrations precisely. A small misalignment would be interpreted as vibrations. Thus, this difference would be removed from the final reconstructed object, provoking inaccurate results. Therefore, it is important to verify that the extracted coordinates from both laser stripes are aligned.

**Figure 13.**
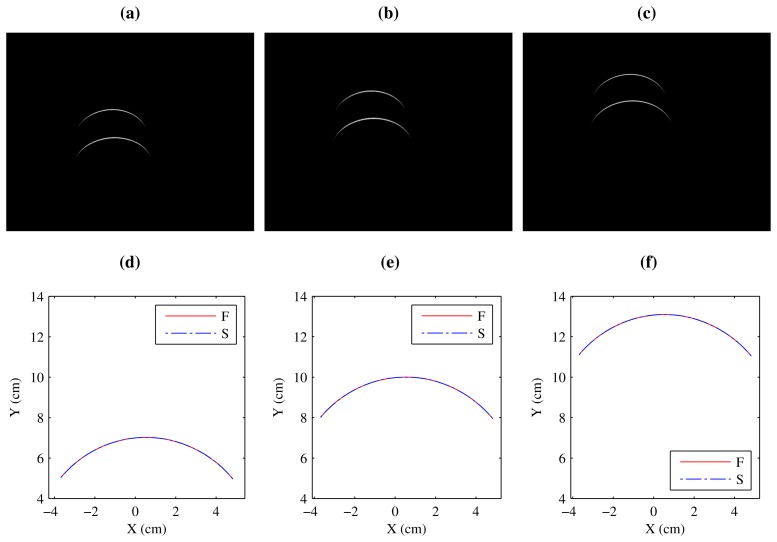
Projection of the laser stripes onto the curved surface. (**a–c**) Image coordinates; (**d–f**) World coordinates.

When the curved surface is used, similar results are obtained, as can be seen in Figure 13. Image coordinates show a clear difference but in world coordinates they are perfectly aligned. In this experiment there is a large occlusion, only the top part of the pipeline is visible in the images. Therefore, in this case only that part would be reconstructed.

**Figure 14.**
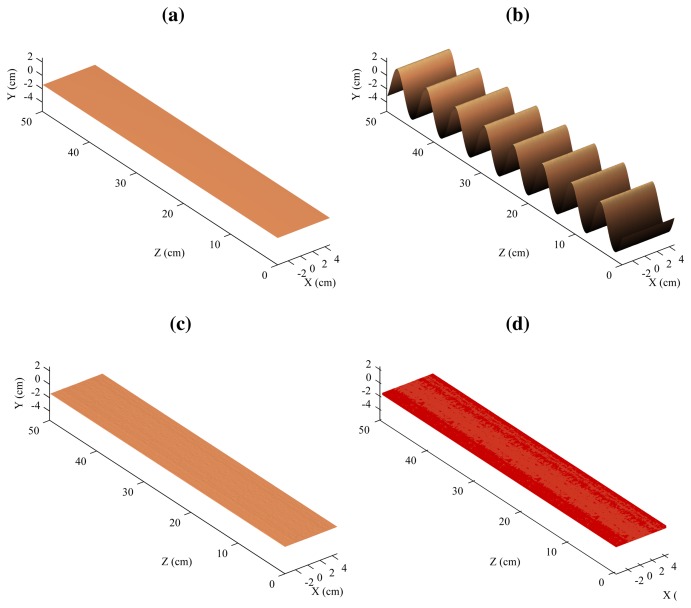
Reconstruction of the flat surface. (**a**) Model; (**b**) Reconstruction using one laser stripe; (**c**) Reconstruction using two laser stripes; (**d**) Model and reconstruction.

The 3D reconstruction of the flat surface can be seen in Figure 14. Figure 14a shows the model, that is, a flat surface. Figure 14b shows the 3D reconstruction using a single laser stripe. In this case, the second laser is ignored. This would be the result using the traditional architecture based a single laser projector. Figure 14c shows the resulting 3D reconstruction when using the proposed procedure based on two laser stripes, and Figure 14d the model and the reconstruction in a single figure.

As can be seen, the reconstruction using a single laser stripe is affected by the vibrations (simulated using the mechanical device). In this case, vibrations are interpreted as part of the surface of the object. However, when the two laser stripes are combined using the proposed approach, the results are very different: Vibrations are removed.

The difference between the model and the reconstructed shape of the object using two laser stripes is assessed with the mean absolute error (MAE) using Equation (29), where *n* is the number of points, *H_i_* is the height at point *i* in the model, and *R_i_* is the reconstructed height at point *i*.

(29) $$\text{MAE}=\frac{1}{\text{n}}\sum_{\text{i}=1}^{\text{n}}\left|H_{i}-R_{i}\right|$$

In the case of the reconstruction of the flat surface, the mean absolute error was 0.066 mm with a standard deviation of 0.046.

**Figure 15.**
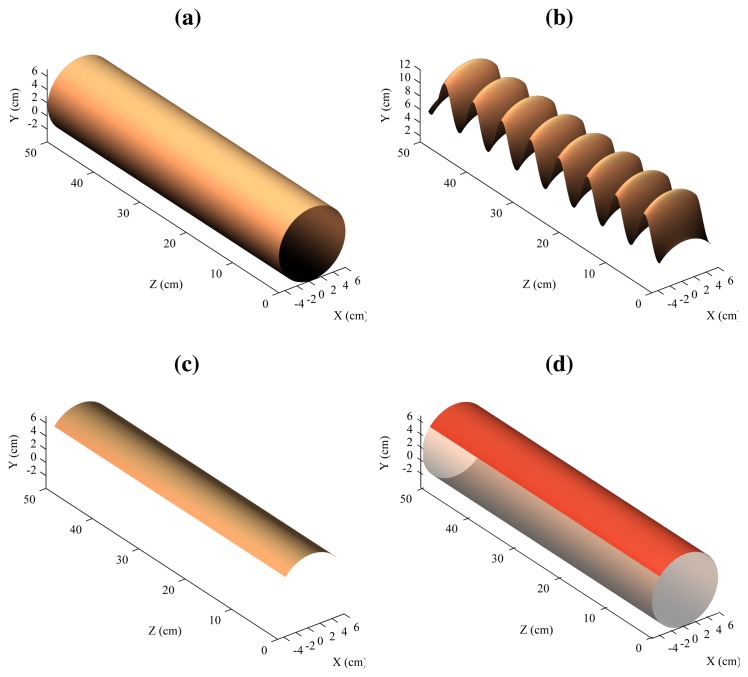
Reconstruction of the curved surface. (**a**) Model; (**b**) Reconstruction using one laser stripe; (**c**) Reconstruction using two laser stripes; (**d**) Model and reconstruction.

The results with the curved surface can be seen in Figure 15, in which the figures show representation equivalent to the flat surface. As can be seen, only the top part is reconstructed due to occlusions. However, this part is a very good approximation of the same in the model. In this case, the mean absolute error was 0.159 mm with a standard deviation of 0.052.

**Figure 16.**
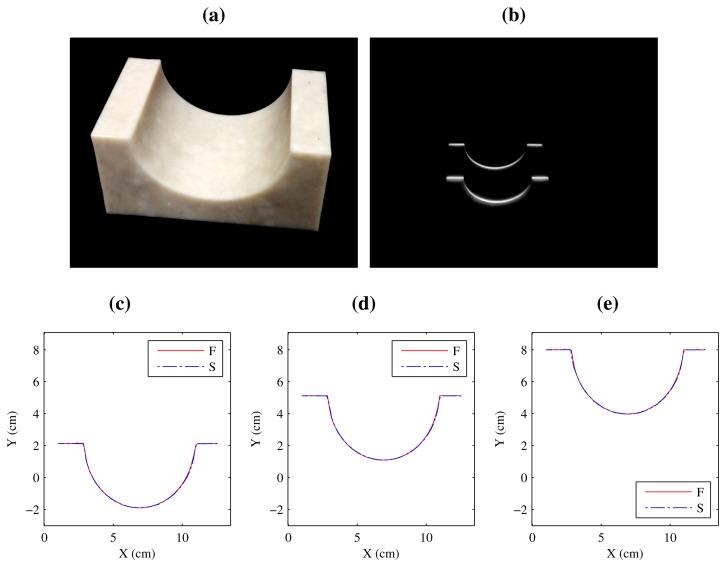
Geometric piece and height profiles. (**a**) Geometric piece; (**b**) Image with the projection of the two laser stripes; (**c–e**) World coordinates of the height profiles while vibrations are introduced.

An additional test has been carried out using a geometric piece that combines flat and curved surfaces. The object can be seen in Figure 16. The laser stripes projected on the piece and the height profiles in world coordinates are shown. As can be seen, height profiles are perfectly aligned while the piece moves up and down due to the simulated vibrations.

**Figure 17.**
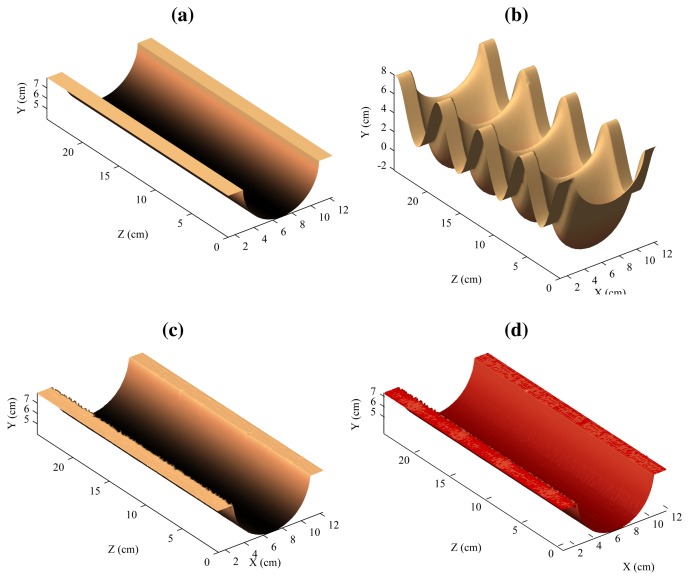
Reconstruction of the geometric piece. (**a**) Model; (**b**) Reconstruction using one laser stripe; (**c**) Reconstruction using two laser stripes; (**d**) Model and reconstruction.

The 3D reconstruction of the geometric piece can be seen in Figure 17. When using a single laser stripe, vibrations lead to an incorrect reconstruction of the surface of the object. However, the redundant information provided by the second laser stripe can be used to remove the effects of these vibrations, as can be seen in the figure. There are some minor errors in the area where the curved surface intersects with the flat surface. This is due to some small occlusions in the projection of the laser stripes, as can be seen in Figure 16b. In this case, the mean absolute error was 0.158 mm with a standard deviation of 0.097, a very similar result to the curved surface.

These results indicate that the proposed procedure is very accurate, removing vibrations almost completely. Shape quality metrics calculated from the reconstruction using only one laser stripe would result in large errors. However, using the proposed procedure, the reconstruction is very similar to the model. Moreover, the calibration setup was very simple and accurate, with no complex calibration objects or elaborated calibration setup.

## 5. Conclusions

3D reconstruction based on laser light projection is greatly affected by vibrations. The movements of the object are interpreted as changes in the extracted height profiles, resulting in significant errors in the reconstructed shape of the object. This work proposes a solution based on two laser stripes. The redundant information obtained in this case can be used to estimate vibrations, and to remove them from the final reconstructed shape. This paper also proposes a method to calibrate a 3D reconstruction sensor with two laser stripes. The proposed procedure has been applied to a real test case with vibrations where both flat and curved surfaces have been reconstructed.

The proposed calibration procedure is very simple and easy to apply. Moreover, it does not require special calibration objects; the same standard calibration plate is used to calibrate both the intrinsic and the extrinsic parameters. This process provides an accurate description of the transformation between the laser planes and the camera, and between the planes, which can be used to extract accurate metric information. The proposed procedure is not limited to two laser stripes; the application to multiple laser stripes is straightforward.

Experimental results show excellent performance. The shape of different surfaces is reconstructed very accurately with vibrations. The redundant information provided by the two lasers can be used to accurately estimate vibrations, and to remove them from the final reconstruction. The reconstructions produced using two laser stripes are a vast improvement over those produced with one.

## Figures and Tables

**Table 1.** Intrinsic camera parameters.

**Intrinsic Camera Parameter**	**Value**
*f*	0.0123
*K*_1_	269.174
*K*_2_	−1.033*e* + 07
*K*_3_	7.776*e* + 10
*p*_1_	−35.944
*p*_2_	33.763
*S_x_*	5.993*e* − 006
*S_y_*	6.000*e* − 006
*C_x_*	637.118
*C_y_*	480.745
